# Leveraging electronic medical record functionality to capture adenoma detection rate

**DOI:** 10.1038/s41598-022-13943-2

**Published:** 2022-06-11

**Authors:** Blake Jones, Frank I. Scott, Jeannine Espinoza, Sydney Laborde, Micah Chambers, Sachin Wani, Steven Edmundowicz, Gregory Austin, Jonathan Pell, Swati G. Patel

**Affiliations:** 1 Division of Gastroenterology & Hepatology, Department of Medicine, University of Colorado School of Medicine, Aurora, CO USA; 2 Rocky Mountain Regional Veterans Affairs Medical Center, Aurora, CO USA; 3 Division of Hospital Medicine, Department of Medicine, University of Colorado School of Medicine, Aurora, CO USA; 4 Division of Gastroenterology & Hepatology, Department of Medicine, University of Colorado School of Medicine, 12631 E 17th Avenue, Room 7614, Campus Box 158, Aurora, CO 80045 USA

**Keywords:** Colonoscopy, Health services, Public health

## Abstract

Measuring the adenoma detection rate (ADR) is critical to providing quality care, however it is also challenging. We aimed to develop a tool using pre-existing electronic health record (EHR) functions to accurately and easily measure total ADR and to provide real-time feedback for endoscopists. We utilized the Epic EHR. With the help of an Epic analyst, using existing tools, we developed a method by which endoscopy staff could mark whether an adenoma was detected for a given colonoscopy. Using these responses and all colonoscopies performed by the endoscopist recorded in the EHR, ADR was calculated in a report and displayed to endoscopists within the EHR. One endoscopist piloted the tool, and results of the tool were validated against a manual chart review. Over the pilot period the endoscopist performed 145 colonoscopies, of which 78 had adenomas. The tool correctly identified 76/78 colonoscopies with an adenoma and 67/67 of colonoscopies with no adenomas (97.4% sensitivity, 100% specificity, 98% accuracy). There was no difference in ADR as determined by the tool compared to manual review (53.1% vs. 53.8%, p = 0.912). We successfully developed and pilot tested a tool to measure ADR using existing EHR functionality.

## Introduction

Widespread adoption of screening colonoscopy has coincided with a steady decline in the incidence of and mortality associated with colorectal cancer in the United States^1^. However, post-colonoscopy cancers do occur and are primarily attributed to missed cancers or pre-cancerous polyps on preceding colonoscopy^2^. Thus, maximizing the quality of endoscopic inspection is essential in reducing missed lesions and post-colonoscopy cancers. The adenoma detection rate (ADR), or the proportion of screening colonoscopies in which at least one adenomatous polyp is found, has become the established surrogate of colonoscopy quality^3^. Multiple studies have demonstrated that performance of ADR correlates with decreased incidence of post-colonoscopy cancers^3,4^ and that providing endoscopists with feedback related to their ADR can improve their ADR performance^5,6^. This has led to the elevation of ADR as a priority quality indicator (QI) and the recommendation that all endoscopists measure their ADR to assure delivery of high-quality and high-value care^7^.

The data needed to determine ADR requires coupling of endoscopic and pathology findings which are separated by both time and location within the electronic health record (EHR). Thus, measuring ADR requires time-intensive chart review either by individual endoscopists or designated staff. Other methods to measure ADR, including the use of natural language processing (NLP) to automatically extract data from endoscopy and pathology text, have been described ^8–14^; though these require complex data management systems that are likely not available to many endoscopists and are not easily transferable from one practice to another^15^. Alternatives to labor intensive manual review and complex NLP based strategies are needed. We therefore aimed to develop and validate a method using embedded EHR functions in Epic, the most widely adopted EHR in the US marketplace^16^, to facilitate both ADR reporting and feedback.

## Materials and methods

**Figure 1:**
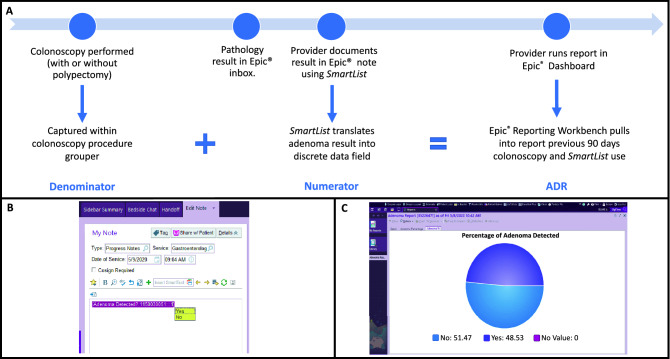
(**A**) Workflow timeline for utilizing the *SmartList* tool. When the report is run, all colonoscopies from the previous 90 days are included and linked to instances of *SmartList* use in the patient’s chart to calculate the tADR in the report. (**B**) The *SmartList* as displayed in a simple note. (**C**) The pie-chart display of the Reporting Workbench results representing the tADR. This figure was constructed from a screenshot from Epic Clinical Software, Version May 2021 (https://www.epic.com/software).

Many EHRs contain functions that allow for automated capture of structed data from EHR text notes^17^. We worked with an Epic analyst to develop a tool using this EHR functionality to capture total ADR (tADR), or the proportion of all colonoscopies regardless of indication in which at least one adenoma is found. Total ADR has previously been shown to correlate with true ADR^18^. The primary Epic functions we utilized were the *SmartList* and Reporting Workbench. A *SmartList* is an item of text that can be embedded in a note to produce data that can be pulled into a report. In addition to the *SmartList*, the build elements necessary for our report included a procedure grouper (an Epic umbrella identifier to capture all the different ways colonoscopies are labeled in the EHR) to identify colonoscopy procedures to the report, a custom Epic property known as results interpreter to interrogate the endoscopist who performed the procedure, and a summary view within the report to display tADR to the endoscopist (Table 1). The *SmartList* was built to record a simple yes or no adenoma response in a note and store it to a *SmartData* element (Table 1, Fig. 1B). The number of yes responses are calculated as a percentage of all colonoscopies and reported as a simple pie chart visible to the endoscopist in the EHR (Fig. 1C). The total count of colonoscopies is determined by looking to the colonoscopy grouper and pulling in all completed procedures for a rolling 90-day lookback, then using the custom results interpreter property to filter the list to the endoscopist(s) of interest thus providing the endoscopist with a real-time tally of their tADR over the last 90 days. Ninety days was chosen to assure that at least 100 colonoscopies were included in each report tally as previous studies analyzing effects of feedback on ADR improvement have utilized at least 100 colonoscopies/provider to assess change^19,20^ The report could be personalized to each endoscopist within our system by changing the name assigned in the results interpreter property as well as the lookback time interval based on endoscopist volume. The components of the report needed to determine tADR are displayed in Table 1.

**Table 1:** Build Components and Report Components required for the Epic based tADR tool.

Build components	***SmartPhrase*** Pre-written text that can be added to a note with a simple dot-phrase
	***SmartList*** Selectable drop-down menu of text options embedded within the *SmartPhrase*
	***SmartData* Element** Selections from the *SmartList* stored as discrete data elements (*SmartText*) that can be prospectively recalled into a report
	**Results Interpreter** A custom Epic build by the analyst to identify and filter the colonoscopy procedure grouper by individual endoscopists
	**Dashboard Component** – A summary of the report’s results displayed in a pie chart format that can be added to an individual providers Epic Dashboard (the screen that is displayed when the provider initially logs into Epic)
Reporting workbench components	**Colonoscopy Procedure Grouper** pre-existing label encompassing the number of different ways the colonoscopy procedure is identified within Epic
	**Results Interpreter** this property was added to the report so that it could be customized to each individual provider. This property along with the grouper served to determine the denominator of the tADR equation
	***SmartData* element results** selections from the *SmartList* stored as either “Yes” or “No” or no response (if there was no polypectomy and thus no indication to utilize the *SmartList*) pulled into the report to determine the numerator of the tADR equation

To minimize workflow the clinical staff were instructed to embed the *SmartList* in a note at time of pathology review, and one provider was selected to prospectively pilot the tool over a 6-month testing timeframe (allowing for 2 90-day Plan Do Study Act (PDSA) cycles). Provider workflow and accompanying EHR functions are displayed in Fig. 1A. Two PDSA cycles were necessary, with iterative expansion of the EHR based colonoscopy grouper until the report captured all colonoscopies performed by our provider over the validation timeframe a greater than 95% sensitivity. A final 90-day audit of colonoscopies performed by the pilot endoscopist was then conducted against a gold standard manual chart review to assess the sensitivity, specificity and accuracy of the report’s ability to capture tADR. Informed consent was waived for this study by the Colorado Multiple Institutions Review Board (COMIRB) because all patient data was de-identified and there was no direct patient contact, nor change in the standard of medical care. This study was reviewed and approved by COMIRB (Protocol Number 19-1378) and was carried out in accordance with all relevant guidelines and regulations.

## Results

One hundred and forty-five colonoscopies were included in the final 90-day validation cohort. A manual chart review found that 78 colonoscopies had at least one adenoma (ADR 53.8%). Of the 145 colonoscopies, our EHR method correctly identified 143 (98.6%). The report identified 76/78 of colonoscopies in which at least one adenoma was found and all (67/67) colonoscopies where no adenoma was found (97.4% sensitivity, 100% specificity and 98.6% accuracy for detection of adenomas). The ADR generated by the EHR report was not clinically or statistically different from the ADR determined by gold standard manual chart review (53.1% vs. 53.8%, p = 0.912). The reason the two colonoscopies with adenomas were not captured by our report is because the endoscopist mistakenly did not utilize the *SmartList* at time of pathology review.

## Discussion

The ADR is a high-value quality indicator because it is readily measured and is an established surrogate for the rare outcome post-colonoscopy cancer risk. Thus, it is imperative that all endoscopists have the means to know and track their ADR, and tools to facilitate more widespread measurement of ADR have the potential to support the delivery of high-value care. Here, we describe a novel method, leveraging pre-existing infrastructure within a widely adopted EHR, to do just that. In a pilot testing environment, our tool demonstrated a high sensitivity and accuracy compared to a manual chart review, similar to what studies evaluating NLP methods report^8–14^. To our knowledge, this is the first description of a method using EHR functionality to accurately capture endoscopic QIs.

There remain weaknesses to the tool we propose. It still requires effort from clinical staff to open a note and utilize the *SmartList* at the time of pathology result documentation. This requires several extra clicks beyond the normal workflow and in our pilot study resulted in 2/78 adenoma positive results being incorrectly classified. Additionally, our tool in its present form does not account for colonoscopy indication, completeness or prep quality. However, as previously noted tADR is an accurate surrogate for ADR, and others have even proposed that it may be a preferred colonoscopy QI as it simplifies measurement and may prevent gaming the ADR metric by changing colonoscopy indication^18,21^. Furthermore, additional macro selections (for instance for indication, bowel preparation quality, polyp histology, such as serrated lesions) can certainly be added to future iterations of our tool. These features can be customized based on a specific practices’ priorities in quality metric tracking and reporting (for instance if this information is not tracked elsewhere). The adaptability and customizability of our tool is a great strength, particularly if professional societies add additional lesion detection rates/benchmarks (such as serrated lesion detection rates) to quality metrics that should be measured.

We believe the tool we propose has several benefits. Because our tool relies largely on pre-existing abilities imbedded within an EHR, it does not require access to specialized data management systems often needed to adopt NLP based solutions. In addition, while NLP methods are often successful at individual institutions, adapting those tools across more diverse clinical settings has proven challenging^15^. Our tool can be scaled for use by anyone using the Epic EHR. Our tool provides real-time feedback within the EHR related to QI performance, allowing endoscopists to confront their own performance in the same interface in which they regularly manage patient care. While our tool does require some minimal effort from clinical staff, this is largely within normal clinical workflow and remains far less than what is required for manual extraction. Finally, though our tool was built using the Epic EHR, multiple EHRs have similar discrete data macro functionalities which could allow a similar tool to be developed in different systems both in the United States and Europe^17,22^.

Further work is needed to validate this tool among a greater proportion of endoscopists and ideally among multiple centers using the Epic EHR. Reassuringly, *Smartlists* are already widely used among everyday documentation in Epic. Additionally, prior research has demonstrated excellent adoption of *Smartlists* in post-colonoscopy EHR documentation^23^. Adjusting the structure of the Reporting Workbench algorithm by utilizing other macro data tools like additional *SmartLists* or flowsheets within the EHR may also allow for capture of additional data such as colonoscopy indication, prep quality and even allow for use of a similar tool to capture QIs in other endoscopic arenas.

This pilot study demonstrates the potential to leverage existing EHR functionality to achieve accurate measurement and feedback of tADR, a reliable surrogate for ADR. This tool may present an easily adoptable alternative to complex NLP based systems or time-intensive chart review to facilitate QI measurement and assure delivery of high-value care.

## Abbreviations

ADR: Adenoma detection rate
QI: Quality indicator
EHR: Electronic health record
NLP: Natural language processing
tADR: Total adenoma detection rate
